# Ohio Dental College

**Published:** 1872-03

**Authors:** 


### Editorial.

### OHIO DENTAL COLLEGE.

The commencement exercises of this Institution were held on Wednesday evening, March 6, in the hall of the College. The exercises were pleasant and interesting, and attended by quite a large assemblage of the profession, and the friends of the Institution. The degree of "Doctor of Dental Surgery" was conferred upon the following persons, viz: Mrs. Marie Grubert, of Berlin, Prussia; B. F. Eager, of Mississippi; C. Shaull, of Ohio; Henry Shelley, Ohio; A. B. Harryman, Indiana; J. H. Warner, Ohio; F. C. Runyan, Ohio; E. B. Fairchild, Kentucky; J. E. Stowers, Kentucky; H. A. Wernett, Ohio; J. P. Morris, Mississippi; J. O. Keller, Ohio.

And upon the following persons the honorary degree was conferred by the Trustees: R. Corson, Middletown, Ohio; W. G. Cummins, Sturgis, Michigan; J. A. Rousey, Toledo, Ohio; Jas. Leslie, Cincinnati, Ohio.

This is the second instance which the degree of D. D. S. has been conferred upon a lady by this Institution, and our experience in this direction is thus far very gratifying indeed. Both stood very well in their respective classes, and both were great favorites with their classmates and with the Faculty. The former has been since her graduation, six years in very successful practice.

The latter, Mrs. M. Grubert, by her courteous manner, dignified bearing, and professional ability, commanded the regard and esteem of all who became acquainted with her. In the Mississippi Valley Dental Society, she received such consideration as has never been bestowed upon any lady before; after being elected to active membership, she was unanimously elected its Vice President. The independence, energy, industry and perseverance which she manifested in the pursuit of her professional studies, were most praiseworthy. She became one of the best operators in her class.

She returns to her native city to enter upon a very promising field of professional labor, which is to be confined exclusively to ladies and children. Many, and urgent were the solicitations she had to remain here; and we have no doubt she would at once have entered upon a large and lucrative practice.

